# Whether the GFR measured by renal scintigraphy under non-steady state conditions for critically ill patients with AKI can be used as a predictive parameter for clinical events

**DOI:** 10.1186/s13054-020-02850-5

**Published:** 2020-04-19

**Authors:** Guang-wen Zhu, Zhou Gao, Abdoul Rachid, Hui Liu

**Affiliations:** 1 Department of Nuclear Medicine, First affiliated Hospital, Dalian Medical University, 222, Zhong Shan Road, Dalian, China; 2 Department of Nuclear Medicine, Peking University Shenzhen Hospital, Shenzhen, China; 3 College of International Education, Dalian Medical University, Dalian, China; 4 ICU of Emergency Department, First affiliated Hospital, Dalian Medical University, Dalian, China

Whether the GFR measured by renal scintigraphy under non-steady state conditions for critically ill patients with AKI can be used as a predictive parameter for clinical events

Dear Editor:

We read with great attention and interest the paper by Katulka et al. about the current evidence for clinical and biochemical parameters for predicting successful discontinuation of RRT [1]. We would like to add some comments in regard to their conclusion that multiple studies reported on the same parameter (urine output, diuretic test, CrCl, cystatin C, NGAL, eGFR, KeGFR), but an optimal threshold value was not determined due also to heterogeneity.

For GFR measurement, we believe that based on the principle of radionuclide-based techniques and its advantages of repeatability of results, the heterogeneity of those above results may be avoided. 99mTc-DTPA has become a standard GFR tracer, the hierarchy of 99mTc-DTPA renal imaging is considered to be the silver standard among all used approaches for detecting GFR [2], including Gates method, modified Gates method, and plasma sample clearance method. Especially for GFR measurement in AKI patients, the result of renal scintigraphy may be more reliable than other existing technologies and is not affected by plasma creatinine changing rapidly, fluid loading in ICU, and receiving CRRT.

To analyze kidney function in the acute setting, recently, our team completed a special renal scintigraphy for a critically ill patient with the only venous access site-PICC. A renal scintigraphy of 99mTc-DTPA was performed for this critical patient with AKI and artificially assisted ventilation. The normalized GFR was 36.5 mL/min (Fig. 1).

**Fig. 1:**
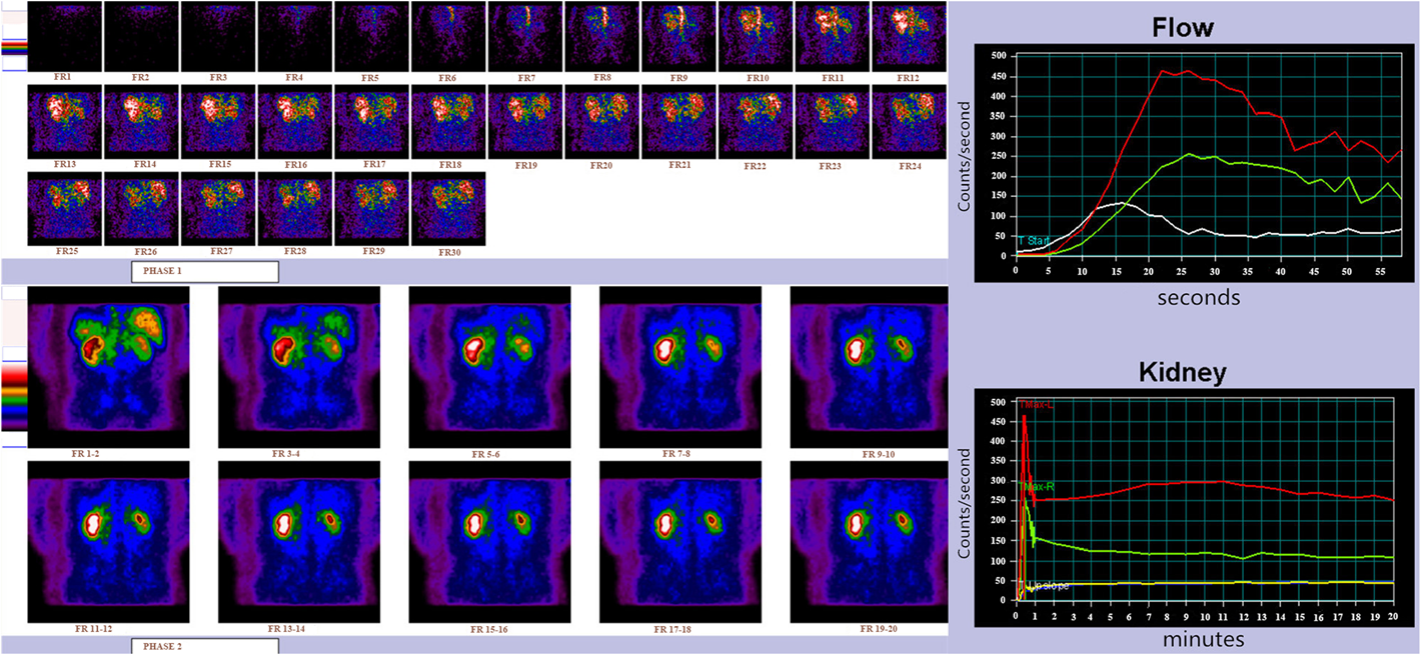
Dynamic renal scintigraphy for a critical patient by PICC. A 65-year-old man with absence of urine for 3 days; he was diagnosed with AKI stage 3, lung infection, atelectasis, electrolyte disorders, and coagulopathy, and the CRRT was initiated. On the fifth day, the patient was performed with endotracheal intubation and ventilator-assisted mechanical ventilation due to dyspnea and hypoxemia. A dynamic renal scintigraphy of 99mTc-DTPA was performed by PICC in this critical patient with AKI and artificially assisted ventilation. The relative renal uptake of the right kidney was 22,737 counts, the left kidney was 40,310 counts, the right kidney GFR was 14.6 mL/min, and the left kidney GFR was 25.9 mL/min. The bilateral renal blood flow significantly reduced and the renogram curves shown a renal failure pattern; the relative renal uptake of each kidney indicated the split renal function, respectively. These findings were most consistent with renal causes of acute renal failure

In the absence of broad guidelines [3], our team proposed the following procedure/practice for renal scintigraphy by PICC/CVC. After intravenous hydration with 300–500 mL of fluid (dextrose; 5% glucose or 0.9% sodium chloride) 30 min, the patency of the catheter in the PICC/CVC system is checked by 10 mL saline solution in a 10-mL syringe. Then, a bolus injection of radiopharmaceutical via PICC or CVC is done, immediately following and flushing with 10–20 mL normal saline or heparin (100 U) sodium solution using the “push-pause” technique for the lock of the system depositing with positive pressure [4], while a standard 20/30-min dynamic renal scintigraphy is performed.

Although renal scintigraphy is rarely used clinically for critically ill patients with AKI, partly because of a lack of understanding of the technology, it can actually provide unique kidneys’ function parameters, split renal function, and GFRs. The GFRs measured by renal scintigraphy alone or with other static and dynamic clinical variables may have a predictive value for stratification and clinical events in critically ill patients.

## Data Availability

Not applicable.

## Abbreviations

GFR: Glomerular filtration rate
AKI: Acute kidney injury
RRT: Renal replacement therapy
CrCl: Creatinine clearance
NGAL: Neutrophil gelatinase-associated lipocalin
eGFR: Estimated GFR
KeGFR: Kinetic eGFR
DTPA: Diethylenetriaminepentaacetic acid
ICU: Intensive care unit
CRRT: Continuous renal replacement therapy
PICC: Peripherally inserted central catheters
CVC: Central venous catheters
